# An Overview of D7 Protein Structure and Physiological Roles in Blood-Feeding Nematocera

**DOI:** 10.3390/biology12010039

**Published:** 2022-12-26

**Authors:** Patricia H. Alvarenga, John F. Andersen

**Affiliations:** Laboratory of Malaria and Vector Research, National Institutes of Health, National Institute of Allergy and Infectious Diseases, Rockville, MD 20852, USA

**Keywords:** D7 proteins, odorant-binding proteins, vector biology, hematophagy, vector saliva, hemostasis, inflammation

## Abstract

**Simple Summary:**

Vectors are organisms that can transmit infectious pathogens from a host (human or animal) to another. Many vectors (including mosquitoes, sand flies and ticks) have one common characteristic: they are blood-feeding (hematophagous) arthropods. Every time they bite their vertebrate host, skin and vascular injury triggers a series of responses that in place could lead to interruption of blood flow to their mouthparts, and to host awareness due to itching and pain. Nevertheless, their saliva contains a cocktail of molecules capable to counteract these host responses (hemostasis, inflammation and immunity), allowing them to feed successfully. Many times, the pathogens they transmit are injected in the host with the saliva. Therefore, understanding the composition of vector’s saliva is crucial to study their biology, their vectorial capacity, as well as to propose new methods to control the diseases they transmit (including new vaccine candidates). Among important salivary protein families is the D7, abundantly expressed in bloodsucking Diptera and distantly related to Odorant-Binding Proteins (OBP). Here, we provide an extensive review of D7 proteins structure, function and evolution, discussing how gene duplication and modifications in their OBP-like domains lead to gain and loss of function in different hematophagous Diptera species.

**Abstract:**

Each time an insect bites a vertebrate host, skin and vascular injury caused by piercing triggers a series of responses including hemostasis, inflammation and immunity. In place, this set of redundant and interconnected responses would ultimately cause blood coagulation, itching and pain leading to host awareness, resulting in feeding interruption in the best-case scenario. Nevertheless, hematophagous arthropod saliva contains a complex cocktail of molecules that are crucial to the success of blood-feeding. Among important protein families described so far in the saliva of blood sucking arthropods, is the D7, abundantly expressed in blood feeding Nematocera. D7 proteins are distantly related to insect Odorant-Binding Proteins (OBP), and despite low sequence identity, observation of structural similarity led to the suggestion that like OBPs, they should bind/sequester small hydrophobic compounds. Members belonging to this family are divided in short forms and long forms, containing one or two OBP-like domains, respectively. Here, we provide a review of D7 proteins structure and function, discussing how gene duplication and some modifications in their OBP-like domains during the course of evolution lead to gain and loss of function among different hematophagous Diptera species.

## 1. Introduction

Vector-borne diseases are quite diverse in terms of symptoms, characteristics, etiologic agents and their vectors. Despite this diversity, a common feature shared by most known vectors is the fact that they are hematophagous arthropods. The ability to feed on blood poses many challenges including the capacity to find the host, pierce its skin and find blood source, suck the blood to later digest it and deal with oxidative stress generated by its digestion (reviewed in [1,2,3]).

Each time an insect bites its vertebrate host, skin and vascular injury triggers a series of responses including hemostasis, inflammation and immunity [1,2,4,5,6,7,8]. These complex and redundant biological processes are interconnected and capable to propagate due to the production/secretion of many classes of mediators, including eicosanoids, biogenic amines, coagulation and complement factors. In place, they would cause interruption of blood flow to the mouthparts of the hematophagous arthropod (due to vascular constriction, platelet aggregation and blood coagulation) and trigger host awareness (due to itching and pain). Nevertheless, the saliva of blood feeding arthropods contains an equally complex and redundant mix of molecules capable to counteract host defenses playing pivotal role in the success of hematophagy (reviewed in [1,2,4,5,6,7,8]). Indeed, as stated before by Ribeiro, hematophagous arthropods are “live syringes” and their saliva are still a relatively unexploited source of pharmacological active molecules [4].

The presence of anticoagulant molecules in hematophagous arthropods saliva was first reported in the beginning of last century [9,10], and the demonstration of the importance of salivary secretion in blood feeding was first proposed in the 80′s [11,12,13]. Yet, the composition of the saliva and the specific function of its components was still poorly understood. The use of large-scale sequencing and proteomics over the last decades allowed a better understanding of saliva composition, but also revealed its complexity and how little is known [2,14]. Because hematophagy has arisen independently several times during arthropod evolution, many ways to deal with the challenges imposed by this habit appeared among the different insect orders, or even within the same genus leading to wide variety in the repertoire of proteins to counteract host defenses to the bite [2,4,5,7,15,16].

Among important protein families described so far in the saliva of blood sucking arthropods, is the D7, abundantly expressed in hematophagous Diptera [17,18,19] and distantly related to insect Odorant-Binding Proteins (OBP)/ Pheromone-Binding Proteins (PBP).

**Figure 1:**
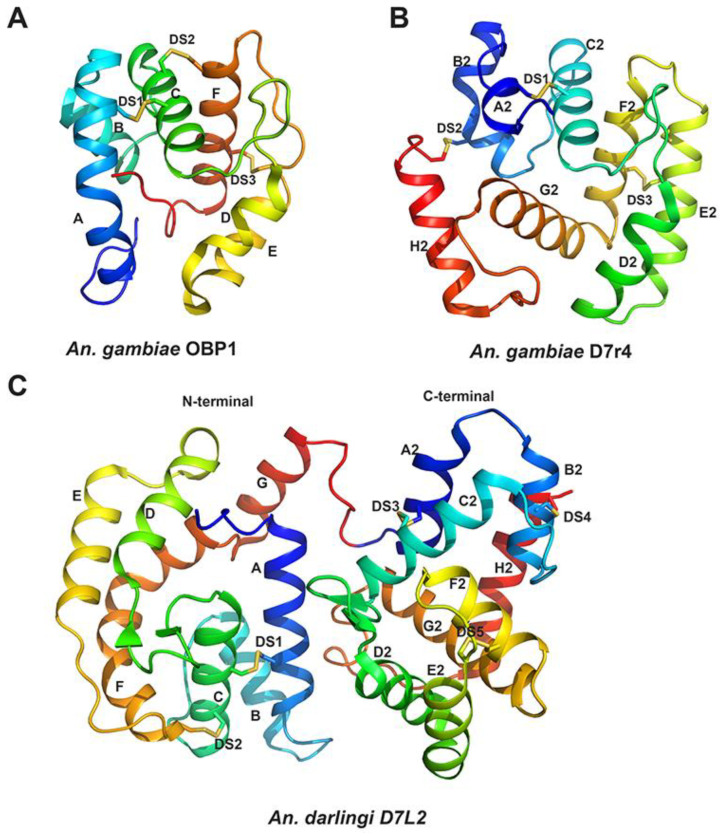
General structure of classical OBPs and D7 proteins—(**A**) Ribbon diagram of *Anopheles gambiae* OBP1 (PDB: 2ERB) [25] representing a classical OBP structure composed by 6 α-helices (labeled A–F) stabilized by 3 disulfide bonds (DS1–DS3) forming a central cavity. Original structure was obtained complexed with PEG. (**B**) *Anopheles gambiae* D7r4 crystallized in its apo form (PDB: 2QEV) [28] representing a D7r protein general structure composed by 8 α-helices (A2–H2) stabilized by 3 disulfide bonds (DS1–DS3). (**C**) *Anopheles darlingi* D7L2 (PDB:7U1N) on its apo form, representing long form D7s, composed by 2 OBP-like domains. N-terminal domain is composed by 7 α-helices (A–G) stabilized by 2 disulfide bonds (DS1–DS2) and C-terminal domains is made by 8 α-helices (A2–H2) stabilized by 3 disulfide bonds (DS3–DS5). In all panels disulfide bonds are shown as yellow sticks, helices are colored by spectrum from blue (N-terminus) to red (C-terminus) of the protein or of the domain (in the case of panel C).

In insects, OBPs typically range from 100 to 160 residues, but as observed for some members belonging to “atypical/two domains” or “Plus C” sub-families described in Diptera, that can have up to 300 amino acids [20,21,22]. They are present in diverse orders and the number of annotated OBP genes vary a lot among different species, as well as the amino acid sequence. Nevertheless, their cysteines are highly conserved—classically 6, although this number can vary from 4 in minus C OBPs to 8 in plus OBPs to even 9 or 10—positioned within the polypeptide chain with a signature distance between some of those [20,21,23]. Differently from vertebrate OBPs, that are all-β proteins belonging to lipocalins superfamily (SCOP: 3001332), insect OBPs are classified as all-α proteins and belong to insect pheromone/odorant-binding super family (SCOP ID: 4000957). Despite the low sequence identity between members, classically insect OBPs have a very characteristic tertiary structure, with 6 α-helices typically stabilized by 3 disulfide bonds forming a binding cavity (Figure 1A), surrounded by hydrophobic residues [20,24,25,26]. The great number and the diversity of their primary structure, while maintaining the overall architecture, made them very versatile in terms of ligands, but all with one characteristic in common: their ability/potential to bind small hydrophobic molecules.

**Figure 2:**
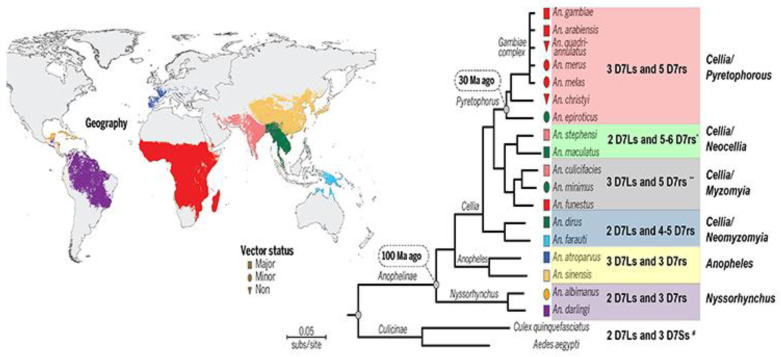
D7 protein family members distribution pattern through Anopheles sub-genus and series and some culicinae species—Geographic distribution and molecular phylogeny (reported by Neafsey et al. [58] using aligned sequence of 1085 orthologs) of the different Anophelinae species whose genomes were sequenced so far. Symbols indicate species vector status and their color matches map color indicating their distribution. In the context of this review we further separated them in sub genus series (indicate on the further right) inside colored boxes. The number of long (D7Ls) and short (D7r or D7S) found in the species of each group [29] as well as in some representative Culicinae species are shown in the colored shaded boxes. In Anophelinae mosquitoes D7L2 and L3 forms are always present, and D7L1 only when a third form is present. In Culicinae mosquitoes the only long forms present are D7L1 and D7L2, while D7L3 was absent in the species analyzed. Short forms observed in Culicinae (*) are shorter, truncated forms reason by which they were not denominated as “related” (D7r), but only as “short” (D7S). Figure modified from: Neafsey, D.E.; Waterhouse, R.M.; Abai, M.R.; Aganezov, S.S.; Alekseyev, M.A.; Allen, J.E.; Amon, J.; Arca, B.; Arensburger, P.; Artemov, G.; et al. Mosquito genomics. Highly evolvable malaria vectors: the genomes of 16 Anopheles mosquitoes. *Science* 2015, 347, 1258522, doi:10.1126/science.1258522. Reprinted with permission from AAAS (License Number 5417800982106).

D7 proteins, nonetheless, are restricted to blood feeding Nematocera, where they are abundantly expressed in their salivary glands. Members of this family are classified in: (1) short forms (D7S), also known as D7 related (D7r), with molecular weight around 15–17 kDa, containing one OBP-like domain (Figure 1B); (2) long forms (D7L), with molecular weights around 30–38 kDa, composed by two OBP like domains (Figure 1C). As first proposed by Arcà and colleagues [27] and later observed by X-ray diffraction crystallography [7,28,29,30], despite their low sequence similarities to OBPs, the D7 protein domains architecture is very similar to OBPs, being composed by α-helices (generally 7–8 α-helices though instead of 6), forming a hydrophobic pocket (Figure 1B,C) suitable to bind hydrophobic molecules, as will be further discussed below.

Most of the D7 proteins characterized so far were found in the saliva of blood sucking Diptera, where they act as anti-hemostatic and/or anti-inflammatory molecules, facilitating blood feeding. In the present paper we provide a comprehensive review of D7 and D7-like proteins structure, function and evolution, comparing some aspects with arthropod OBPs and other protein families relevant to vector physiology.

## 2. D7s: From the Description of the First Gene to Clues Regarding Their Diversity, Distribution and Functions

The first gene encoding a D7 protein was isolated and described in 1991 in *Aedes aegypti* [31], shown to be abundantly and exclusively expressed in female salivary glands producing a 37 kDa protein. In situ hybridization using dissected female salivary glands probed with antisense RNA for the coding region of this gene revealed its expression was mainly in the distal lateral and medial lobes, regions that are very well differentiated in females (hematophagous) when compared to males (phytophagous), suggesting its product was very likely to be involved in blood feeding. Later, in a pioneering study aiming to identify genes that were expressed exclusively in the salivary gland of *Anopheles gambiae* and whose products contained signal peptides, six cDNAs were isolated. Three of them, shown to be abundantly expressed in female salivary glands, encoded transcripts similar, albeit shorter, to the previously described *Aedes aegypti* D7, suggesting that this was a new protein family. All three aligned completely with *Aedes* D7 C-terminal domain, therefore being named D7-related proteins (D7r): D7r1–D7r3 [32]. Likewise, they were expressed in female distal lateral lobes, although only one of them (D7r1) was expressed in medial lobes too [32]. A few years later, a fourth D7r (D7r4) was also found in *Anopheles gambiae* female salivary glands and shown to be located close to the other three forms forming a cluster on chromosome 3R [27]. When aligned, these 4 transcripts had similarity between them ranging from 53 to 73%, and very importantly, they had low level of similarity to OBPs and Pheromone binding Proteins (PBP), but their 4 cysteines were located at conserved positions related to antennal and non-antennal OBPs. Despite low sequence similarity, secondary structure prediction suggested structural similarities to OBPs, hence it was postulated that D7s might also have a hydrophobic binding pocket surrounded by α-helices and be capable to bind or carry small hydrophobic molecules, probably mediators involved in host responses (like inflammation and hemostasis), given the fact that they were abundant and exclusive to female saliva [27].

The fact that these *An. gambiae* short forms aligned with the C-terminal of the *Ae. aegypti* D7L, and that Southern blot assays found other members in closely related species raised the possibility that members of this gene family might be present in other mosquito species, and that proteins with different lengths encoded by these genes could have similar functions, but different targets due to difference in their primary structure [27].

Indeed, Suwan and coworkers reported for the first time genes encoding one D7L and 2 D7-related (short) forms in the salivary gland of *Anopheles stephensi*, and Western blot with polyclonal antibody produced to recognize the long form protein had cross reactivity also with D7rs [33]. This was the first report showing that indeed long and short forms could be found in the same species. Then, subsequent studies reported the presence of other members of this family in different mosquito (family Culicidae) species including *Anopheles arabiensis*, *Aedes aegypti* and *Anopheles darlingi* [17,18], as well as in other hematophagous Diptera belonging to the family Psychodidae (sand flies) [17].

Altogether these studies strongly suggested that: (1) D7 was a family of proteins probably widespread in hematophagous Diptera, exclusively expressed in female salivary glands, therefore they might have an important role in blood feeding; (2) D7 proteins different in length and amino acid sequence were present in different species and within the same species, probably as a result of gene duplication generating diversity; (3) their putative targets should be small hydrophobic molecules, such as inflammation and/or hemostasis mediators [27,33]. Nevertheless, their function was still elusive.

## 3. Salivary D7 Proteins Are Anti-Inflammatory and Anti-Hemostatic Molecules

D7 and other OBP like proteins present in the saliva of blood sucking Nematocera can have variability in their primary structure, leading to gain and loss of function despite keeping some key features and general architecture, like has been observed to many OBPs. Table 1 summarizes D7 and D7-like proteins described so far in the saliva of different species, their ligands and provides their Protein Data Bank (PDB) accession number, where applicable. Details and physiological relevance are discussed below.

### 3.1. Contact Pathway Inhibitors

The first D7 to have its function characterized was a short form (D7r), named Hamadarin [35], expressed in the SG of *Anopheles stephensi* females. Its closest D7 expressed in *An. gambiae* would be D7r1. Hamadarin was shown to bind Factor XII (FXII) and High Molecular Weigh Kininogen (HMWK) therefore inhibiting contact pathway activation and consequently bradykinin production, acting as an anti-inflammatory molecule. Nevertheless, in both cases the interaction with FXII and HMWK, studied by Surface Plasmon Resonance (SPR), occurred only in the presence of Zn^2+^ and had no effect on activated factors amidolytic activity. Rather, its inhibitory effect on contact pathway activation was due to its interference on reciprocal activation of FXII and kallikrein that should occur upon their interaction with charged surfaces [35]. A few years later another contact pathway activation inhibitor present in the saliva of *Anopheles stephensi* (Anophensin) was characterized and shown to have similar targets to Hamadarin, but belonged to a completely different protein family [42].

Noteworthy, among the most expressed proteins in sandfly saliva [43,44,45], is a group of proteins that also belong to OBP superfamily, are not found in mosquitoes and are distinct from D7 proteins. They first got attention not only for their abundance, but because they were identified as vaccine candidates against Leishmaniasis [46,47]. Only more than a decade later two members of this group were characterized in the saliva of *Phlebotomus duboscqi*: PdSP15a and PdSP15b (*P. duboscqi* Salivary Protein 15 a and b, respectively). Both are very similar to each other and inhibit contact pathway activation and bradykinin production [41], but through a mechanism distinct from that described for Hamadarin [35]. PdSP15a and b do not bind to any coagulation factor (zymogens or activated forms), kallikrein or prekallikrein. Their action though is through binding negatively charged polymers such as dextran sulfate (DS), polyphosphate (PP) and heparin, preventing their interaction with FXII, necessary for its auto-cleavage that triggers its activation, and subsequent propagation of contact pathway activation through reciprocal activation of FXII and plasma pre kallikrein (PK), as well as activation of FXI by thrombin and FXIIa. Importantly while classical OBPs and D7s have a hydrophobic pocket where their small hydrophobic ligand binds, structural data suggest its mechanism of action is through electrostatic interaction between its anionic surface and negatively charged ligands, rather than any binding inside a hydrophobic cavity [41,47].

The plasma coagulation cascade can be initiated by two distinct branches: (1) the extrinsic pathway, initiated when factor VII (FVII) is activated upon binding to subendothelial tissue factor (TF) exposed after vascular injury. (2) The contact pathway, also known as the intrinsic pathway, is initiated upon activation of FXII to FXIIa triggered by its contact with negatively charged surfaces. Both pathways after a series of reactions converge to the common pathway through FX activation that ultimately leads to fibrinogen cleavage to fibrin, essential for clot formation [48,49]. In recent years, drugs targeting intrinsic (contact) pathway components such as FXII and FXI have been extensively studied since it was shown that mice lacking FXII were protected from thrombus formation while having no major bleeding disorders [50,51,52], suggesting this pathway is important for pathological coagulation.

Upon activation, factor XII cleaves plasma pre kallikrein (PK) generating kallikrein, that in addition to reciprocal activation with FXII also catalyzes the hydrolysis of HMWK generating bradykinin (kallikrein-kinin system) [53]. Bradykinin is a potent pro inflammatory mediator that increases endothelial permeability [48,54] and pain [55]. Therefore, the presence of contact pathway inhibitors in the saliva of hematophagous arthropods would play an important role as anti-inflammatory molecules by reducing bradykinin production (as shown for the D7s/OBP-like proteins hamadarin [35] and PdSP15s [41]), and by inhibiting/preventing plasma leakage induced by contact pathway activation as reported for PdSP15s [41].

### 3.2. Biogenic Amine Binding D7s

There are more than 460 species of *Anopheles*, divided in 7 subgenera [56]. So far, the genome of 18 species of *Anopheles* (reference strains) have been sequenced and are available in Vector Base, representing the three main medically important subgenera of *Anopheles*: *Cellia*, *Anopheles* and *Nyssorhynchus* that occupy different regions in the globe and diverged from each other up to 100 million years ago (as is the case between *Cellia* and *Nyssorrhynchus*) [57,58,59]. *Anopheles sp* mosquitoes whose genomes were published so far (Figure 2) have 2–5 D7S (depending on the subgenera and series), plus at least two long forms (D7L2 and D7L3), while some have a third long form (D7L1, present in some *Cellia* series and in *Anopheles* subgenus) [29,60].

A few years after Hamadarin’s characterization, the 5 D7rs (D7r1-D7r5) whose transcripts were previously observed in *Anopheles gambiae* (*Cellia*) female salivary glands [27,32] were characterized [34]. All of them, except D7r5, were reported to bind serotonin with very high affinities (dissociation constants, K_D_, below 3 nM) as well as histamine with K_D_ ranging from 41 to 111 nM. Curiously, but not surprisingly their ability to bind other biogenic amines and their affinities for them was also distinct (summarized in Table 1), suggesting divergence of function among different members of D7 family, even within the same species. In all the cases, the binding stoichiometry was 1:1 and competition assays suggested that biogenic amines share the same binding site [34], as later confirmed by the crystal structure of *Anopheles gambiae* D7r4 bound to serotonin and other biogenic amines [28].

Orthologs of *An. gambiae* D7rs are found in all Anophelines analyzed species, whose genomes are annotated in Vector Base so far (Figure 2), although some lost one or more members [29,60]. Regardless of the variation on the sequences throughout the different species and groups, critical residues lining the biogenic amines, identified thanks to *Anopheles gambiae* D7r4 structural data [28], are extremely conserved in practically all D7r1-D7r4 forms across species belonging to subgenera *Cellia, Nissorhynchus* and *Anopheles*, suggesting they all retained the ability to bind serotonin [29]. On the other hand, in all species D7r5 show alterations in various critical residues [29] suggesting that like observed experimentally for *Anopheles gambiae* D7r5 [34], they lost the ability to bind any biogenic amine.

Curiously, while the Anopheline D7S forms seem to have in general conserved their biogenic amine binding capacity, this is not true for the long forms, where lots of variation, neo functionalization and loss of function are observed across the species belonging to the different sub genera.

The first *Anopheles* D7L to be characterized, originally named *Anopheles stephensi* D7L1 (AnSt-D7L1), now considered a D7L2 due to its similarities to *An. gambiae* forms, was shown to be unable to bind serotonin or any biogenic amines tested, but bound eicosanoids in its N-terminal domain [30]. Like *Anopheles gambiae*, *An*. *stephensi* belongs to the sub genus *Cellia*. Nevertheless, recently D7L members belonging to other Anopheline sub genera *An. atroparvus* D7L1 (*Anopheles*) and *An. darlingi* D7L2 (*Nyssorhynchus*) were shown to bind serotonin with very high affinity (in their C-terminal domain) [29], displaying K_D_s comparable to the observed for *Anopheles gambiae* short forms [34] and *Aedes* long forms [34,37,38]. Nonetheless, in general their capacity to bind other biogenic amines was absent or considerably lower. The loss of ability to bind serotonin observed in *An. stephensi* can be better understood thanks to structural data [30].

**Figure 3:**
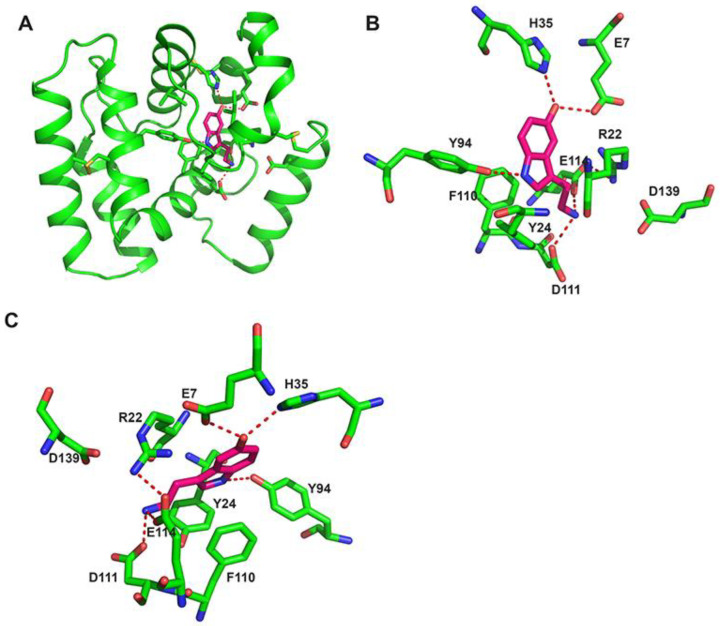
Details of *Anopheles gambiae* D7r4 biogenic amine binding pocket—(**A**) Ribbon diagram of An. gambiae D7r4-serotonin complex (PDB:2QEH). (**B**,**C**) Two different views showing details of *An. gambiae* D7r4 bound to serotonin (PDB:2QEH) [28]. Protein helices and residues forming the biogenic amine binding pocket (labeled with one letter code and position) are shown in green, and serotonin is in hot pink. Cysteines forming the 3 disulfide bonds (DS 1–3) are also shown and labeled. Oxygens atoms are colored in red, nitrogen atoms in blue and sulfur in yellow. H-bonds are indicated as red dashed lines.

**Figure 4:**
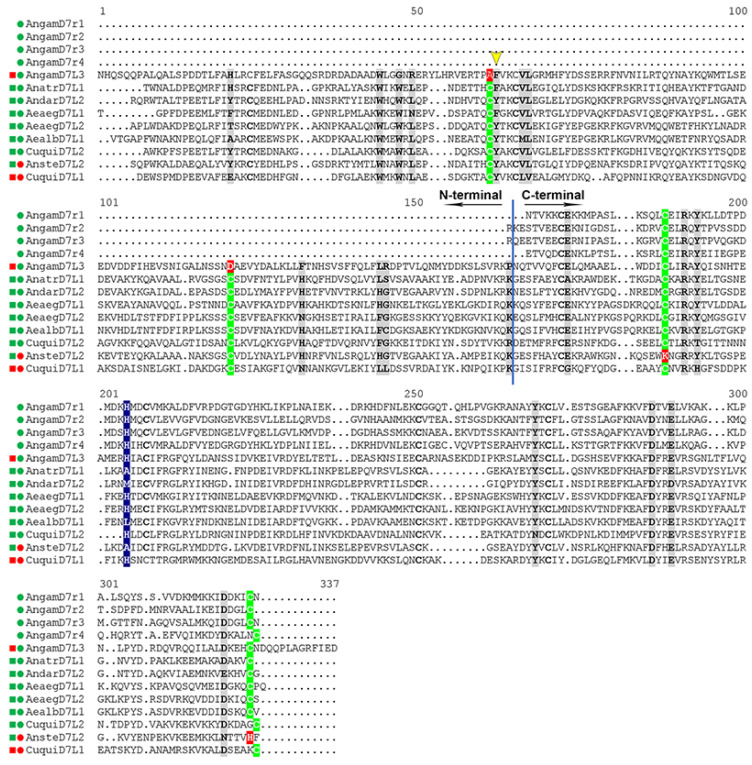
Alignment of D7 proteins characterized so far in different species of mosquitoes whose ligands were identified experimentally—Sequence comparison of some short (D7S or D7r) and long (D7L) forms of D7 characterized so far. Circles before each species name represent the ability of short forms and long forms C-terminal OBP like domain to bind biogenic amines (in this generalization, serotonin); squares represent the ability of long forms N-terminal domain to bind eicosanoids (cysteinyl leukotrienes). The filling color of the symbol represent whether they bind (green) or not (red) the respective ligand. N and C-terminal domains are separated by a blue line and indicated in the figure. Residues lining the binding pockets with relevance to the ligand-binding interactions as commented in the text are in grey boxes. Cysteines involved in disulfide bonds are in bold, and the ones in key positions, are highlighted in green boxes. The absence of these in some members (red box in the position) instead of green is one of the factors that might have led to loss of function, as is the case for AnSt-D7L1 (here named as *An. stephensi* D7L2) C-terminal that does not bind biogenic amines. In some biogenic amines binding D7s, a histidine (position highlighted in a blue box) can form a hydrogen bond with the 5-hydroxyl of serotonin further stabilizing the ligand, this is not crucial, since members having an alanine and a methionine in this position were shown to bind too, in a way that is independent of this interaction [29]. In eicosanoid binding long forms, when tyrosine is present instead of a phenyl-alanine in the position indicated by a yellow arrow head the proteins gained the capacity to bind TXA_2_ in addition to cysteinyl leukotrienes.

In Anopheline mosquitoes, D7S (or D7r) proteins align to the C-terminal domain of *Anopheles* and *Aedes* D7L, and X-ray crystallography studies confirmed that D7S proteins and the OBP-like C-terminal domain of biogenic amine binding D7L are very similar structurally [28,29,36]. As a general characteristic, the serotonin/biogenic amine binding pocket is a hydrophobic cavity lined with aromatic groups, surrounded by 8 α-helices that are stabilized by 3 disulfide bonds (Figure 1B and Figure 3). The presence of some polar charged residues in the entrance of the pocket (glutamic and aspartic acids) allow hydrogen bonds with the aliphatic part of serotonin (Figure 3 and Figure 4). The ligand is further stabilized by a hydrogen bond formed between its indole group and a tyrosine (Tyr 94 in *An. gambiae* D7r4 Figure 3). Residues known to be important for serotonin/biogenic amine binding are highlighted in gray boxes in Figure 4. Alignment shows that most of them are conserved, despite the difference in the other residues, even in proteins shown not to bind biogenic amines, like *An. stephensi* D7L1, where the loss of binding is due to a few residue modifications (Figure 4).

In some proteins, the serotonin 5-hydroxyl group forms a hydrogen bond with a histidine (Figure 3 and Figure 4 highlighted with a blue box) as observed in D7r4 and *Aedes aegypti* D7L1 (His 35 in the first and His 189 in the second). This His is substituted by an alanine (Ala-190) in An-StD7L1, but this would not be enough to explain the loss of function observed, since *An. darlingi* D7L2 (subgenus *Nyssorhynchus*) and *An. atroparvus* D7L1 (subgenus *Anopheles*) bind serotonin even though this His is substituted by a methionine or an alanine, respectively [29]. Therefore, the critical difference distinguishing *Anopheles stephensi* D7L1 from mosquito D7s that bind serotonin is the loss of the second and the last cysteine in their C-terminal domain (green and red boxes C-terminal domain, Figure 4). These two residues would form the second disulfide bond of the C-terminal, the fourth of the whole protein, therefore labeled as DS4. In their absence there is a shift of helix H2 and unwinding of helix B2, as a result W173, that by the way is not present in biogenic amine binding D7s, and R177 (AnSt-D7L1) occupy part of the binding pocket, explaining its inability to bind biogenic amines, as shown and discussed in detail previously [30].

**Figure 5:**
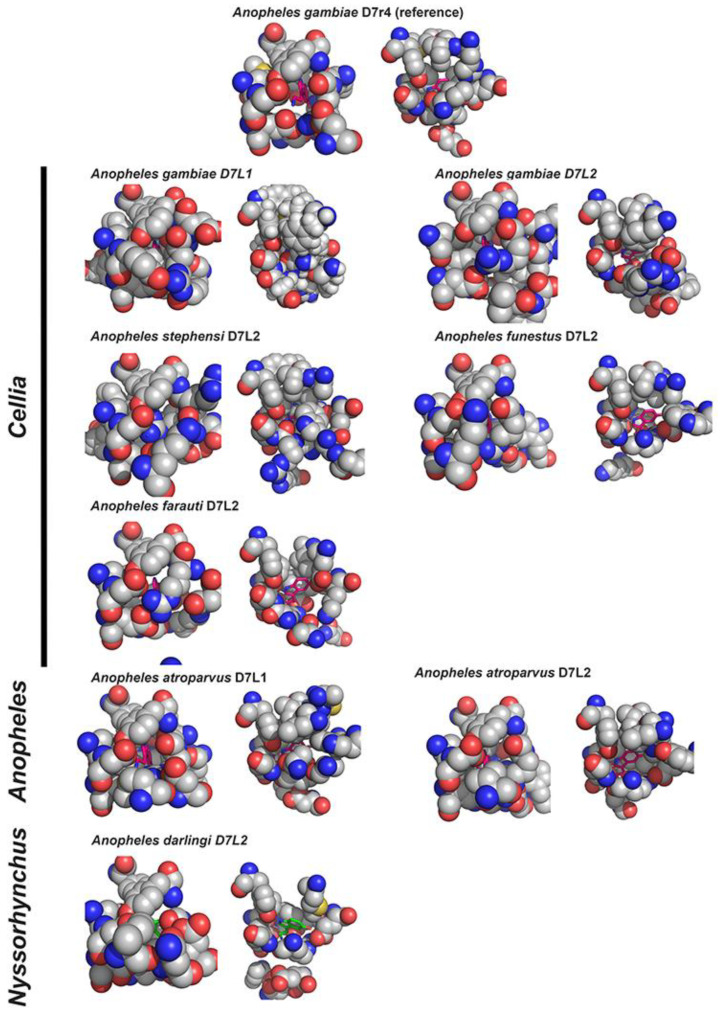
Space -filling model representation of *An. gambiae* D7r4 biogenic amine binding site and C-terminal domain of D7L1 and D7L2 proteins from *Anopheles* species representing distinct sub genera and series—Solved structure of *Anopheles gambiae* D7r4 bound to serotonin (PDB: 2QEH) was used as a reference, and D7L2 and D7L1 (when present) structures (modeled or solved) were aligned to the first. Residues lining the binding pockets were assigned and their atoms (hydrogens are omitted) are represented as spheres proportional to their atomic radii, colored by element (carbons in grey spheres, oxygens in red, nitrogen in blue and sulfur in yellow). Proteins are shown in 2 different views, taken from each protein in the exact same position obtained from superimposed aligned structures. Serotonin is represented in pink sticks, kept from solved D7r4 structure aligned as a reference of the space it would require in each pocket, except in *Anopheles darlingi* D7L2, where serotonin is represented in green and comes from data collection of the protein co-crystallized with it (PDB: 7TX8).

The absence of these two cysteines is also observed in all D7L1s and D7L2s expressed in all species belonging to subgenus *Cellia* leading to the suggestion that they might have lost the biogenic amine binding function as well [29]. This hypothesis is further supported by the observation of their models, constructed using AlphaFold [61] (Figure 5). Like the observed experimentally for *An. stephensi* D7L1 [30], the absence of DS4 in *Cellia* D7L1 and D7L2 proteins is accompanied by a shift in helix H2 position and other structural rearrangements that lead to a bulkier C-terminal pocket with residues occupying part of the cavity not leaving enough space to accommodate serotonin or other biogenic amines (Figure 5). On the other hand, the degree of unwinding in helix B2 observed depends on the species, or might be a result of a more unstable helix transitioning from one state to the other.

Another group of long forms is D7L3, present in all *Anopheles* species with available genome so far. *Anopheles gambiae* D7L3 binds serotonin with high affinity and specificity [29], and its C-terminal domain has all the critical amino acids shown to be involved in biogenic amine interaction [28] conserved (Figure 4) and with the same spatial disposition as D7r4 [29]. This conservation was also observed in all D7L3s analyzed from *Anopheles* species, regardless of the subgenera, suggesting that this form, that lies in an adjacent position to short forms in the D7 clusters, conserved this function in all these species [29].

In Culicinae mosquitoes, D7S orthologs and 2 D7Ls (D7L1 and D7L2) are also found. Nevertheless, as opposed to the observed in Anophelinae, their short forms do not bind biogenic amines accordingly to results reported for *Aedes aegypti* (AeD7S1) and *Culex quinquefasciatus* (D7CQS1) [29], while their long forms characterized so far, with exception of *Culex quinquefasciatus* D7L1, have very high affinity for those ligands (specially serotonin) as reported for *Aedes aegypti* D7L1 (previously named AeD7L) and D7L2 [34,37], *Ae. albopictus (D7L1)* [38] and *Culex quinquefasciatus* (CxD7L2) [39]. Structural data suggest that the loss of function in their short forms, despite containing all the 6 conserved cysteines and being composed of α-helices, is due to a shortening in their C-terminal lacking α-helix H2 region, and consequent differences in the α-helices arrangements that lead to blockage of the binding pocket [29]. Very importantly, culicines lack D7L3, the long form that in *Anopheles* sp. lies immediately adjacent to the short forms in the cassette, and as most of the DS (D7r1–r4) in *Anopheles*, binds serotonin [29].

Biogenic amines are mediators of diverse processes involved in vertebrate responses to bites, many times interconnecting them [6,62]. Histamine, for example is a potent mediator of inflammatory and allergic responses and is released by mast cell degranulation. It activates the endothelium, increases vascular permeability and promotes itching and pain [63,64,65,66,67]. It is also known to promote smooth muscle contraction. Serotonin and norepinephrine promptly released by activated platelets and neutrophils are agonists of platelet aggregation and vasoconstriction. Serotonin is also involved in inflammatory response by activating the endothelium and promoting itching and pain [6]. The effectiveness and significance of these D7s for vector biology was shown by their ability to inhibit smooth-muscle contraction [34,37] induced by different biogenic amines and the interfering with serotonin mediated platelet activation [38].

### 3.3. Eicosanoid Binding D7s (the N-Terminal Domain of D7Ls)

The fact that D7L forms have two OBP-like domains raised the possibility that these proteins could harbor other ligands in their N-terminal domain. Indeed, *Ae. aegypti* D7L1, known to bind biogenic amines [34], was the first long form shown to bind cysteinyl leukotrienes (CysLTs) and leukotriene B4 (LTB4) by its N-terminal [36]. Soon after, *An. stephensi* D7L1 (AnSt-D7L1), that does not bind biogenic amines or any other tested ligand in its C-terminal domain, was shown to bind not only CysLTs with extremely high affinity, but also thromboxane A2 (TXA_2_) analogs (U46619 and carbocyclic thromboxane) [30]. Isothermal titration calorimetry (ITC) assays and structural data showed that both ligands share at least part of the binding site located in its N-terminal domain [30].

Other D7L1 and L2 proteins in Culicinae and Anophelinae mosquitoes were also shown to bind eicosanoids, but with different affinities and specificities, in addition to their capacity to bind serotonin. In Culicinae (*Ae. aegypti* D7L2 [37], *Ae. albopictus* D7L1 [38] and *Cu. quinquefasciatus* D7L2 [39]) were shown to bind CysLTs and LTB4 (the later only in *Aedes*), but with affinities significantly lower than reported for *Ae. aegypti* D7L1. Interestingly, they acquired the ability to bind U46619. In Anophelinae mosquitoes. *An. atroparvus* D7L1 (*Anopheles*) binds CysLTs, but with low affinity, while *An. darlingi* D7L2 (*Nyssorhynchus*) binds CysLTs with very high affinity and TXA_2_ analogues (U46619) [29]. These D7s proteins are bi-functional, since they also bind serotonin.

Comparison of binding site residues from ligand complex crystals from *An. darlingi* D7L2, *An. stephensi* D7L1 and *Ae. aegypti* D7L1 [29,30,36] enabled the assignment of critical residues for eicosanoid binding at N-terminal domain (highlighted in Figure 4). Of particular importance for the stabilization of the ligands are Trp-37, Trp-40 and Tyr-52 (AnSt-D7L1 as reference), and when the latter is substituted by a Phe (yellow arrowhead Figure 4), as observed in *Ae. aegypti* D7L1, the ability to bind TXA_2_ in addition to CysLTs is lost. Equally important is the presence of Lys-152 forming a hydrogen bond or salt bridge with the carboxyl of the eicosanoid. Many of these key residues are conserved in D7L1s and D7L2, especially in the latter, present in other Anophelinae species analyzed, suggesting that regardless of the sub-genus they conserved at least one long form (D7L1 and/or D7L2) capable of binding cysteinyl leukotrienes [29], as shown more recently to be the case of *Anopheles gambiae* D7L1 and D7L2 [68]. These two *An. gambiae* D7Ls were unable to bind biogenic amines but preserved the ability to bind eicosanoids [68], not a surprise given their similarity (specially D7L2 form) to previously described *Anopheles stephensi* D7L1 (now L2) [30] and that both species belong to sub genus *Cellia* and their D7L1 and L2 lack the DS4 on the C-terminal domain.

Noteworthy, while D7L3s are present in all *Anopheles* species and have their residues linked to serotonin binding at C-terminal extremely conserved, the N-terminal of all species were predicted to be unable to bind eicosanoids due to significant substitutions in residues that are known to be important for this task. This hypothesis was confirmed experimentally when ITC had shown that *An. gambiae* D7L3 does not bind any eicosanoid tested [29].

Very interestingly, D7L forms are also found in sand flies (family: Psychodidae) [17,69] despite the evolutionary distance between them and mosquitoes (Culicidae). Long forms characterized in the saliva of two different species of *Phlebotomus* (*P. papatasi* and *P. duboscqi*) retained the capacity to bind CysLTs with extremely high affinities and TXA_2_ analogs [40]. Structural data obtained from *P. papatasi* D7L1 have shown that the eicosanoid binding occurred also in the N-terminal, in a similar way as described for mosquitoes’ D7L, while its C-terminal was shorter and truncated, therefore unable to bind biogenic amines [40].

Observations of fossils suggest that the first Diptera appeared in the Triassic, more than 240 million years ago (MYA). By the end of Triassic, Culicomorpha and Psychodomorpha infraorders appeared, meaning that mosquitoes and sand flies lineages diverged more than 200 MYA, very likely from a phytophagous ancestor, suggesting that they developed the habit to feed on blood independently [5,70]. This is supported by the fact that most of the salivary gland protein families that are exclusively found in Nematocera are different between Psychodidae and Culicidae families, meaning that is very hard to assign orthologs between them [5]. Therefore, mosquito and sand flies D7Ls, probably originated independently from a similar or common ancestral gene, likely coding for an OBP, that was later recruited independently to their sialome and gained that function by convergent evolution. This hypothesis is further supported by the observation that they have different intron/exon structure [40].

The absence of D7S orthologs in sand flies and the inability of their D7L orthologs to bind biogenic amines does not mean their saliva lack molecules to sequester these targets. Indeed, in sand fly saliva another protein family “Yellow” has taken over the function to bind biogenic amines [71], while the other family of OBP-like protein PdSP15 found in their saliva acts by inhibiting contact pathway activation [41]. This is a great example of how independent evolution leads to different repertoires of proteins targeting the same molecules.

Leukotrienes (CysLTs and LTB4) are potent inflammation and allergy mediators secreted by activated mast cells and other immune cells such as eosinophils and macrophages, as well as epithelial and endothelial cells [72]. CysLTs have been shown to be released as a response to mosquito bites together with histamine [62], causing increased vascular permeability in the skin [63] and consequent erythema and wheal formation [73,74], while LTB4 is known as a chemoattractant responsible for attracting immune cells to the site of response [72]. The ability to bind these potent pro-inflammatory mediators would be important to inhibit endothelium activation, edema formation, immune cell infiltration, itching and pain triggered by these eicosanoids, therefore preventing or delaying host awareness and allowing these insects to feed on blood. This anti-inflammatory effect was shown in mouse models when injection of *Ae. albopictus* D7L1 (that binds LTB4, CysLTS, and biogenic amines, in addition to low affinity for U46619) 10 min prior to pro-inflammatory challenge with β-glucan from *Sacharomyces cerevisae* reduced immune cells influx into the peritoneal cavity [38].

Thromboxane A_2_ is produced and secreted by activated platelets in response to collagen exposure. It then binds to its receptors present on the platelet surface propagating platelet activation and potentiating aggregation [75,76]. Very importantly, in addition to TXA_2_ platelets also secrete other pro-hemostatic and proinflammatory molecules, such as ADP, serotonin, polyphosphate and norepinephrine [6]. TXA_2_ also promotes vasoconstriction [77,78] and more recently was shown to elicit itching and scratching responses in mice [79,80].

Several D7Ls were shown to bind U46619, a more stable TXA_2_ analogue by ITC experiments and shown to inhibit platelet aggregation in vitro induced by U46619. Importantly, these proteins also inhibited platelet aggregation induced by arachidonic acid (thromboxane A_2_ precursor) and lower concentrations of collagen (in which platelet aggregation relies on TXA_2_ and ADP to potentiate the signal), proving that they indeed are capable to bind platelet synthesized TXA_2_, and not only its stable analogue used for ITC and crystallography experiments [29,30,37,38,40].

CysLTs and TXA_2_ also are known to promote smooth muscle contraction. Assays have shown that *An. stephensi* D7L1 (now classified as a D7L2), for example, was able to inhibit LTC4 promoted guinea pig ileum contraction and U46619 promoted rat aorta contraction in vivo [30].

Insect OBPs were originally described in olfactory and gustatory appendages, where they would bind, solubilize and transport semiochemicals, as well as regulate the duration of odorant response. Later they were shown to be present also in non-sensory organs, such as midgut, accessory glands, testis, seminal receptacle, Malpighian tubules and even in wasp venom gland, indicating they might have a broad range of ligand and their functions are not restricted to chemoreception (reviewed in [20,21]). Therefore, most of the binding assays and structure data available were performed with ligands such as pheromones, odor molecules, alcohols and other synthetic organic compounds [20,26,81,82,83,84]. No insect OBP was shown to bind biogenic amines so far. Nevertheless, some OBPs were shown to bind long chain fatty alcohols, like bombykol, a pheromone produced by *Bombyx mori* [82], or long chain fatty acids and arachidonic acid, precursor of eicosanoids as reported for *Aedes aegypti* OBP22 [85,86], for example. *Ae. aegypti* OBP22 is present in the antenna, female proboscis and male reproductive organs and is transferred to females during mating [86], suggesting its function is not restricted to chemoreception. Structural studies show that in ligand-free state this protein is composed by 6 α-helices like insect classical OBPs. However, in the presence of ligand, OBP22 undergoes a conformational change in its C-terminal forming a seventh α-helix [85] enlarging the binding pocket. Noteworthy, authors observed that this OBP has highest similarity to the N-terminal domain of D7L proteins, and its seventh helix formed upon binding to fatty acids occupies a very similar position to the seventh helix observed in these D7L lipid-binding domains [85].

### 3.4. ADP Binding D7s

*Culex quinquefasciatus* D7L1 (CxD7L1), differently from any D7L characterized so far lacks ability to bind eicosanoids or biogenic amines, probably due to a few but important modifications in some critical positions at their N- and C- terminal pockets, such as the presence of a glycine instead of glutamic acid in position 155, that is very important to form a hydrogen bond with 5-hydroxyl group of the serotonin indole ring in the majority of biogenic amine binding D7s, as well as a histidine at position 172 instead of a tyrosine, as observed in the majority of biogenic amine-binding D7 proteins (Figure 4). Instead, it was shown to bind adenosine phosphorylated derivatives ATP, ADP and AMP (5′adenosine tri-, di and mono diphosphate, respectively) with high affinity, adenosine and adenine with significantly lower affinities [39]. Another peculiarity is the fact that the interaction with its ligands occurs between the N- and C-terminal OBP-like domains, rather than cavities inside any of them [39].

ATP and ADP intracellular concentrations are tightly maintained and when there is any injury, ADP and ATP are released in the extracellular milieu following cell lysis and can act as pro inflammatory and pro hemostatic molecules [76,87]. ADP activates platelet aggregation and is secreted by activated platelets in response to agonists, such as collagen exposed after vascular injury, to further propagate aggregation [76,88]. Due to its ability to bind ADP, CxD7L1 [39] was shown to inhibit platelet shape change induced by lower concentrations of collagen, as well as aggregation triggered by higher doses (1 μM) of ADP and lower doses of collagen in which aggregation depends on the secretion of second mediators such as ADP and TXA_2_.

## 4. Mosquito Juvenile Hormone-Binding Protein (mJHBP): What Is a D7-like Protein Doing in Mosquito Hemolymph?

In 2017 Kim and colleagues [89], in an effort to find D7-related proteins expressed outside salivary glands, found and described a new protein primarily present in the hemolymph of pupae and adults (male and female) *Aedes aegypti* mosquitoes. Orthologues of this protein were also found in different *Anopheles* and *Culex* species, sharing more similarity than their salivary D7 proteins. Like salivary D7 long forms, this protein has two OBP-like domains. Its N-terminal conserved many of the residues shown to be involved in eicosanoid binding in salivary D7s, suggesting a lipid binding pocket could be present, while its C-terminal composition was very different from any other D7/D7-like described so far. ITC experiments have shown that this protein, named mosquito Juvenile Hormone-Binding Protein (mJHBP), lacks the ability to bind eicosanoids but has high affinity and specificity for juvenile hormone (JH). Structural data show that indeed its N-terminal domain architecture is similar to their counterparts in D7L proteins, containing two disulfide bonds and being composed by 7 α-helices and contains most of the residues involved in its interaction with JH III. Nevertheless, differently from observed for salivary D7L proteins described so far, some of the C-terminal residues also participate in the binding, especially the extension of helix α-13 closing the entry of the binding pocket [89]. Very importantly, as well addressed by the authors, this protein is structurally completely different from the hemolymph juvenile hormone binding protein described so far in *Bombyx mori* [90].

Juvenile hormone regulates the most diverse processes in insects including development [91], molting and metamorphosis [92], reproduction and oogenesis [93,94,95] and immunity [96,97]. When the physiological role of *Aedes aegypti* mJHBP was studied by knocking its gene out by CRISPR-cas9, no effect on development, growth or reproduction was observed [98]. Nevertheless, knocked out (KO) mosquitoes had impaired innate immune response, being more susceptible to bacterial infection when challenged with sub-lethal doses of *E. coli* and producing significantly lower amounts of antimicrobial peptides following infection, when compared to wild type (WT) mosquitoes. These effects were consistent with lower number and different composition of hemocytes in KO mosquitoes observed by the authors [98].

## 5. Conclusions

Salivary OBP-like proteins, like D7 and PdSP15 family members play crucial role facilitating blood feeding, targeting different molecules involved in host hemostasis and inflammatory response. Gene duplication of salivary genes, including D7s, and rapid mutation lead to gain and loss of functions within different family members. This diversity is not exclusive of D7 proteins and has been described in other families such as insect lipocalins [6,7].

Host defenses to bite are not exclusive to hematophagous Diptera, nor are the target or the presence of proteins to address them. Nevertheless, the way to overcome these challenges is diverse among arthropods groups due to independent evolution of hematophagy resulting in a wide repertoire of proteins to counteract host hemostatic, inflammatory and immune responses [2,4,5,7,15,16]. For example, D7 proteins can bind biogenic amines and eicosanoids. In ticks and triatomine bugs lipocalins (independent evolution), a completely different protein family with very distinct architecture composed of 8 antiparallel β-sheets surrounding a binding pocket, takes over these functions [7,99,100,101,102,103,104,105]. In sand flies, short form D7s are absent, and biogenic amine binding function is taken over by “yellow” protein family [71], while they have D7Ls that bind eicosanoids [40].

*Culex quinquefasciatus* D7L1, differently from other D7s, binds ADP. Apyrases, enzymes that catalyze the hydrolysis of ATP and ADP to AMP and Pi (inorganic phosphate) as well as ADP binding proteins and 5′nucleotidases were described in the saliva of diverse hematophagous arthropods species, not to mention other proteins that inhibit platelet aggregation targeting other molecules such as collagen and thrombin (reviewed by [76]).

Understanding the composition of saliva is crucial for the study of vector biology and their interaction with the host. In addition, it provides valuable information to the development of new vector-borne disease control approaches. For example, in most vector borne diseases the pathogen is injected in the host together with vector saliva during the bite. The fact that some salivary proteins are immunogenic, make them great epidemiological tools as biomarkers for human exposure to vector bite, as reported for *Aedes* salivary gland protein extract [106] and *An. gambiae* D7s [107]. Their ability to elicit immune responses also make them great vaccine candidates, as is the case of PdSP15, a OBP like salivary protein shown to be a promising vaccine candidate against cutaneous leishmaniasis [47].

## Figures and Tables

**Table 1:** D7s and OBP-like proteins characterized in Nematocera, respective ligands and PDB accession number (when available).

Protein (Accession Number)	Ligands (KD in nM, When Available)	PDB	References
*An. gambiae* D7r1 (AGAP008284)	Serotonin (1.99)		[34]
	Histamine (103)		
*An. gambiae* D7r2 (AGAP008282)	Serotonin (2.93)		[34]
	Norepinephrine (2.84)		
	Histamine (90)		
	Epinephrine (64)		
*An. gambiae* D7r3 (AGAP008283)	Serotonin (0.16)		[34]
	Norepinephrine (3.19)		
	Histamine (41.0)		
	Epinephrine (312.0)		
*An. gambiae* D7r4 (AGAP008281)	**	2QEV	[28,34]
	Serotonin (0.93)	2QEH	
	Norepinephrine (645)	2QEO	
	Histamine (111)	2QEB	
	Tryptamine	2PQL	
*An. gambiae* D7r5 (AGAP008280)	No binding to any biogenic amine tested		[34]
*An. stephensi* D7r1 (hamadarin) (ASTE016512)	Factor XII		[35]
	High Molecular Weight Kininogen		
*Aedes D7S1* *(AAEL006406)*	No binding to any biogenic amine tested	7TVC	[29]
*Cu. quinquefasciatus* D7S (D7CQ1) (AAR18437.1)	No binding to any biogenic amine tested	7TVY	[29]
*An. gambiae* D7L3 (AGAP028120)	Serotonin (22.2)		[29]
	Histamine (1536.1)		
	Norepinephrine (2531.6)		
	No binding to tryptamine, octopamine, dopamine or epinephrine		
	No binding to any eicosanoid tested or ADP		
*An. stephensi* D7L2 (previously D7L1) (AF420266.1)	**	3NGV	[30]
	LTC4 (3.7)	3NHI	
	LTD4 (5.0)		
	LTE4 (6.1)		
	U46619 (98.0)	3NHT	
	Carboxyclic thromboxane A2 (38.3)		
	PGD2 (1500.0)		
	PGE2 (1900.00)		
	PGF2a (671.0)		
	U51605 (775.0)		
	No binding to any biogenic amine tested, LTB4 or ADP.		
*An. atroparvus* D7L1 (AATE004070)	Serotonin (2.9)		[29]
	Tryptamine (<2)		
	Histamine (548.8)		
	LTC4 (257.0)		
	LTD4 (460.8)		
	LTE4 (344.8)		
	No binding to dopamine, octopamine, norepinephrine or epinephrine		
	No binding to U46619 or any other eicosanoid tested		
*An. darlingi* D7L2 (EU934268.1)	**	7U1N	[29]
	Serotonin (17.5)	7TX8	
	Tryptamine (518.1)		
	Dopamine (833.3)		
	Octopamine (1113.5)		
	Norepinephrine (3257.3)		
	LTC4 (13.8)		
	LTD4 (26.1)		
	LTE4 (243.9)		
	U46619 (91.7)	7TX8	
	No binding to histamine, epinephrine or LTB4		
*Ae. aegypti* D7L 1 (AAEL006424)	**	3DXL	[34,36]
	Serotonin (0.39)		
	Norepinephrine (0.119)	3DYE	
	Histamine (140)		
	Epinephrine (102)		
	LTC4 (57.4)		
	LTD4 (54.3)		
	LTE4 (60.2)	3DZT	
	LTB4 (140)		
	No binding to U46619		
*Ae. aegypti* D7L2 (AAEL006417)	Serotonin (1.68)		[37]
	Norepinephrine (110)		
	Histamine (1130)		
	LTC4 (5270)		
	LTD4 (597)		
	LTE4 (1930)		
	LTB4 (769)		
	U46619 (69.4)		
	No binding to epinephrine		
*Ae albopictus* D7L1 (AALF024478)	Serotonin (4.51)		[38]
	Norepinephrine (3.67)		
	Histamine (278)		
	Epinephrine (4110)		
	Dopamine (11)		
	Tryptamine (570)		
	LTC4 (67.7)		
	LTD4 (332)		
	LTE4 (567)		
	LTB4 (342)		
	U46619 (946)		
*Cu. quinquefasciatus* D7L1 (CPIJ014549)	5’-ATP (30.77)		[39]
	5’-ADP (32.68)	6V4C	
	5’-AMP (77.52)		
	Adenosine (312.50)		
	Adenine (1760.56)		
	No binding to 5’-GTP, 5’-TTP, 3’-AMP, cAMP or PolyP		
	No binding to any biogenic amine or eicosanoid tested		
*Cu. quinquefasciatus* D7L2 (CPIJ014551)	Serotonin (7.46)		[39]
	Histamine (383.14)		
	Epinephrine (226.24)		
	LTC4 (151.75)		
	LTD4 (156.49)		
	LTE4 (158.73)		
	U46619 (934.58)		
	Arachidonic acid (1083.42)		
	No binding to U46619, 5’ATP or 5’-ADP		
*P. papatasi* D7L (AGE83092)	LTC4 (5.88)		[40]
	LTD4 (1.75)		
	LTE4 (15.87)		
	U46619 (751.8)		
	No binding to any biogenic amine tested		
*P. duboscqi* D7L (ABI15936)	**	6MTF, 6MT7	[40]
	LTC4 (3.2)		
	LTD4 (4.16)		
	LTE4 (29.4)		
	U46619 (1282.0)		
	No binding to any biogenic amine tested		
*P. duboscqi* Salivary protein 15 a and b PDSP15a (ABI15933) PDSP15b (ABI15943)	**	4OZD	[41]
	Polyphosphate		
	Dextran sulfate		
	Heparin		
	No binding to FXII, FXIIa, FXI, prekallikrein or kallikrein		

Data regarding ligands for each protein characterized so far and binding affinities (when available) were retrieved from original manuscripts cited in the table. When structural data is available for a given protein co-precipitated with its ligands, respective PDB accession number is provided in the same line as the ligand. ** Structural data was obtained from proteins in the absence of the ligands.

## Data Availability

Not applicable.
